# Synchronous abdominal tumors: is combined laparoscopic surgery in a single approach a safe option?

**DOI:** 10.1590/S1677-5538.IBJU.2017.0429

**Published:** 2018

**Authors:** Marcelo Cartapatti, Roberto Dias Machado, Roberto Lodeiro Muller, Wesley J. Magnabosco, Alexandre César Santos, Brian Francis Chapin, Armando Melani, Antonio Talvane, Marcos Tobias-Machado, Eliney Ferreira Faria

**Affiliations:** 1 Hospital de Câncer de Barretos, Barretos, SP, Brasil; 2 MD Anderson Cancer Center, Houston, TX, USA; 3 Faculdade de Medicina do ABC, Santo André, SP, Brasil

**Keywords:** Laparoscopy, Neoplasms, Neoplasms, Multiple Primary

## Abstract

**Background and Purpose:**

Recent advances in cancer treatment have resulted in bet- ter prognosis with impact on patient's survival, allowing an increase in incidence of a second primary neoplasm. The development of minimally invasive surgery has provided similar outcomes in comparison to open surgery with potentially less mor- bidity. Consequently, this technique has been used as a safe option to simultaneously treat synchronous abdominal malignancies during a single operating room visit. The objective of this study is to describe the experience of two tertiary cancer hospitals in Brazil, in the minimally invasive treatment of synchronous abdominal neoplasms and to evaluate its feasibility and peri-operative results.

**Materials and Methods:**

We retrospectively reviewed the data from patients who were submitted to combined laparoscopic procedures performed in two tertiary hospitals in Brazil from May 2009 to February 2015.

**Results:**

A total of 12 patients (9 males and 3 females) with a mean age of 58.83 years (range: 33 to 76 years) underwent combined laparoscopic surgeries for the treatment of at least one urological disease. The total average duration of surgery was 339.8 minutes (range: 210 to 480 min). The average amount of intraoperative bleeding was 276.6mL (range: 70 to 550mL) and length of hospitalization was 5.08 days (range: 3 to 10 days). Two patients suffered minor complications regarding Clavien system during the immediate postoperative period.

**Conclusions:**

Combined laparoscopic surgery for the treatment of synchronous tumors is feasible, viable and safe. In our study, there was a low risk of postoperative morbidity.

## INTRODUCTION

Recent advances in cancer treatment have resulted in an improvement of prognosis with a profound impact in patient's survival (1). This fact, associated with the advances in technology and diagnostic methods, have led to an increase in the incidence of a second primary neoplasm. The published frequency of multiple malignancies is 1 to 3% according to international literature (1, 2). While diagnosis of synchronous primary tumors is relatively uncommon, their management warrants consideration. Experts prioritize the treatment of the most aggressive malignancy with the worst prognosis (3).

Surgical treatment of synchronous tumors of the same histologic origin has been routinely performed in many oncologic scenarios (1–4). Usually, it consists in extraction of a primary tumor and a solitary metastasis, as in colon cancer. A systematic review published in 2009 showed that, in selected cases, patients with a colon primary tumor and synchronous liver metastasis could be treated by open surgery in a single procedure, instead of two surgeries as a sequential treatment, with the same feasibility and complication rates (4).

The benefits of minimally invasive treatment in contemporary urologic practice are well established. Similar oncologic outcomes are achieved in comparison to open surgery, however with potentially more desirable cosmetic results, less post-operative pain, shorter hospital stay, lo-wer complication rates and earlier postoperative recovery (5–7). Since the first laparoscopic ne-phrectomy published by Clayman et al. in 1991 (8), there have been numerous technological de-velopments that have allowed a wide adoption of minimally invasive procedures for the treatment of both benign and malign urologic diseases.

Because of the well tolerated minimally invasive surgical approach, performing simultaneous treatment of synchronous abdominal malignancies in a single procedure has been accepted by the international urological society (9). Due to the lack of extensive data on combined laparoscopic surgeries, current literature fails to demonstrate the perceived benefits and efficacy of this approach. The objective of this study is to describe the experience of two tertiary cancer hospitals in Brazil, in the minimally invasive treatment of synchronous abdominal neoplasms and to evalua-te its feasibility and peri-operative results.

## MATERIALS AND METHODS

We retrospectively reviewed the data from patients who underwent combined laparoscopic procedures performed in two tertiary hospitals in Brazil from May 2009 to February 2015. All In-formed consents were applied before each surgery and were not repeated for data collection.

The inclusion criteria for the study group were as follows: (A) both procedures were performed laparoscopically under a single application of anesthesia; (B) at least one of the procedures was performed for the treatment of a urologic disease and (C) the procedures were in different sites of the abdomen, meaning two separate procedures. Specialists in oncology and minimally invasive surgery, in an inter-disciplinary approach, performed all the surgeries. The initial procedure was elected according to physician's decision and in agreement with both surgeons. All procedures were performed under general anesthesia. Bowel preparation was performed only for colorectal surgeries and antimicrobial prophylaxis for all procedures was done with a first-generation cephalosporin. In case of colorectal surgery, it was added metronidazole. Postoperative complications were described and classified according to the Clavien-Dindo Classification of Surgery Complications (10).

The data was obtained from patient records (emergency room data, visits and office) and patient demographics were recorded. Descriptive statistical analyses were made using the software IBM SPSS Statistics version 20. All ethical aspects of this manuscript were reviewed and approved by the Institution Review Board from both institutions.

## RESULTS

A total of 12 patients: 9 males (75%) and 3 females (25%) with an average age of 58.83 years (range: 33 to 76 years) underwent combined laparoscopic surgery for the treatment of at least one urologic disease. The most common type of urologic surgery, which was performed on 10 patients, was to address a renal tumor (partial or radical nephrectomy and nephroure-terectomy). Additionally, one patient underwent an adrenalectomy and one had a radical prostatectomy. The combined laparoscopic procedures consisted of 10 non-urological procedures (6 colectomies, 3 rectosigmoidectomies and 1 total gastrectomy), while 2 had synchronous urologic procedures (left nephroureterectomy with right partial adrenalectomy and left radical nephrectomy with right adrenalectomy). All the procedures performed and their respective pathological findings are listed in Table-1.

**Table 1:** Pathological findings regarding urologic and non-urologic diseases.

Patient	Pathology test Urological pathology	Pathology test Non-urological pathology
1	Anaplastic plasmacytoma	Anaplastic plasmacytoma
2	High grade urothelial carcinoma	Colon adenocarcinoma
3	Renal cell carcinoma (clear cells)	Moderately differentiated colon adenocarcinoma
4	Adenocarcinoma arising from tubulovillous adenoma	Metastatic adenocarcinoma (adrenal)
5	Renal cell carcinoma (clear cell)	Tubular adenocarcinoma of the rectum
6	Renal cell carcinoma (clear cell)	Mucinous gastric adenocarcinoma / chronic cholecystitis
7	Adenocarcinoma metastasis / poorly differentiated adenocarcinoma	Poorly differentiated adenocarcinoma
8	Renal oncocytoma	Rectum tubulovillous adenocarcinoma
9	Renal cell carcinoma (clear cell)	Adrenal carcinoma
10	Renal cell carcinoma (clear cell)	Colon Adenocarcinoma
11	Urothelial carcinoma	Adrenal adenoma
12	Prostate adenocarcinoma, Gleason 4+4	Well -differentiated adenocarcinoma

**Figure 1:**
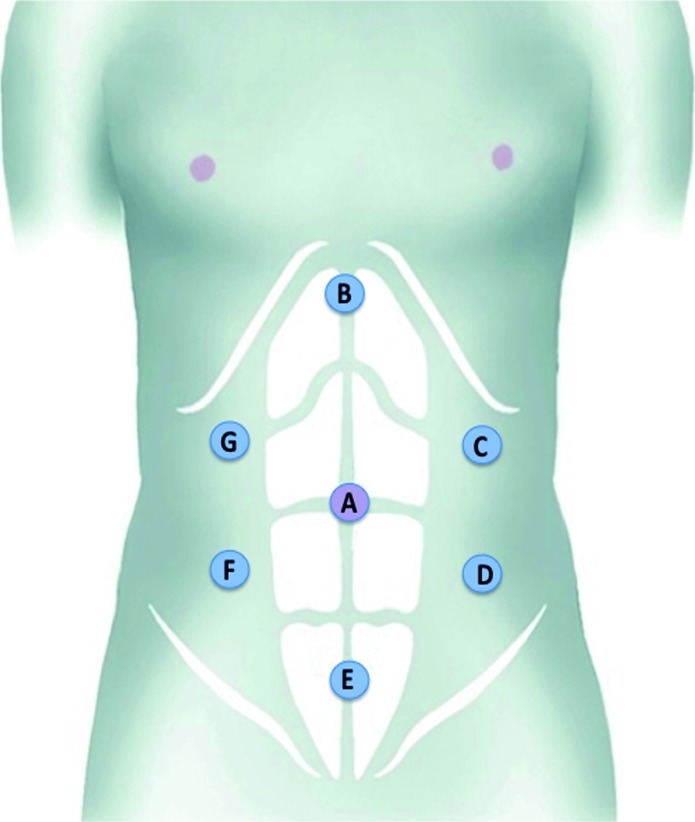
Port Placement.

The mean duration of surgery was 339.8 minutes (range: 210 to 480 min.). The average amount of intraoperative bleeding was 276.6mL (range: 70 to 550mL). All procedures were performed using a trans-peritoneal approach with number of trocars varying from a minimum of 4 to a maximum of 6. The number of trocars utilized was minimized whenever possible and placement was coordinated between surgeons to accommodate their individual needs. The schematic figure provides the suggested port placement for all cases (Figure-1). The specimens were placed in a laparoscopic specimen bag and removed at the end of the procedure by Pfannenstiel, median infra umbilical or Gibson's incision, depending on patient position or surgeon's preferences. There were no intraoperative complications, no conversions to open surgery and no intraoperative mortalities in this group. The average length of hospitalization was 5.08 days (range: 3 to 10 days). Only two patients suffered complications during the 90 days postoperative period; one patient had a transitory hand paresthesia related to surgical positioning and the other one was diagnosed with nosocomial pneumonia and treated successfully with empirical antibiotics. Both complications were classified as Clavien II, and were considered minor. The remainder of patients experienced no complications during the 90 days postoperative period. Table-2 summarizes the demographic and intraoperative data.

**Table 2:** Demographic caractheristics, intraoperative data and complications.

Patient	Age (y)	Gender	Urologic Surgery performed	Non-urologic Surgery performed	Time (min.)	EBL (mL)	IOC	LOH days	POC
1	33	M	Left radical Nephrectomy	Total colectomy	350	150	No	6	No
2	75	F	Right radical nephrectomy	Left colectomy	470	400	No	7	No
3	55	M	Right radical nephrectomy	Right colectomy	210	200	No	3	No
4	74	F	Left adrenalectomy	Right colectomy	358	200	No	5	No
5	57	M	Right Partial Nephrectomy	Rectum amputation	360	550	No	4	No
6	76	M	Left radical nephrectomy	Subtotal gastrectomy + cholecystectomy	290	500	No	7	No
7	53	M	Right radical nephrectomy	Right hemicolectomy	300	70	No	3	No
8	53	M	Right Partial nephrectomy	Recto-sigmoidectomy	480	350	No	5	No
9	50	M	Left radical nephrectomy	Right adrenalectomy	250	150	No	2	No
10	60	F	Right Partial Nephrectomy	Right colectomy	370	200	No	5	No
11	65	M	Left Nephroureterectomy	Right partial adrenalectomy*	220	150	No	10	Yes
12	55	M	Radical Prostatectomy	Rectosigmoidectomy	420	400	No	4	Yes

**IOC** = intraoperative complications; **POC** = postoperative complications; **LOH** = Length of Hospitalization

## DISCUSSION

The American Cancer Society has recently reported that 20% of Americans will develop cancer in their lifetime. Furthermore, for those patients who develop a tumor, the chance of developing a second tumor during their lifetime is around 30% (3). Specific to urologic cancers, the finding of a second primary tumor has been reported in the literature with an incidence range of 3.3 to 6.6% (11–14). A subset of these patients will develop two primary tumors synchronously, leaving the oncologists with a difficult dilemma: which tumor should be treated first (1–3)?

In the urology field, the finding of a second primary tumor has been reported in the literature. Sugiyama et al. (11) found an incidence of multiple cancers in their patients with urologic cancer of 6.6%. In another study by Wegner et al. (12), over a 19-year period at the University of Berlin Hospital, there was a secondary tumor incidence of 3.3%. Nogueras-Gimeno et al. (13) and Mikata et al. (14), in two other studies, found an incidence of 6.1% and 6.4% of secondary tumors in their patients with urologic cancer, respectively.

Simultaneous open surgery has been described as a successful treatment option for other synchronous non-urological malignancies (4, 15). Hillings et al. (4) performed a meta-analysis of studies comparing combined versus staged resection of synchronous liver metastases from colorectal cancer, focusing on length of hospital stay, in-hospital morbidity, mortality and 5-year survival. The data analysis revealed that combined surgery may lead to a shorter length of hospitalization and less morbidity, but it does not seem to affect long-term survival, and in the early decade, at least, it had a larger 30-day mortality. Kim et al. (16) were one of the pioneers to demonstrate that patients with synchronous abdominal tumors could undergo laparoscopic surgery in a single procedure. In a series of 10 laparoscopically managed cases of metastatic colon cancer with liver implants, the results regarding intra and post-operative com-plications and length of hospitalization were comparable to published series that describe these procedures carried out separately (17).

With the advance of new technologies, laparoscopy became a safe and effective treatment option for various urological diseases with many potential benefits. Patients may experience a shorter hospitalization period, early postoperative recovery and better cosmetic results (9, 18). Therefore, specialists have sought to extend these benefits to combined laparoscopic procedures for the treatment of two or more diseases. This strategy has numerous advantages for patients such as: exposure to a single anesthesia; reduction of hospitalization; potential for decreased morbidity and postoperative pain due to smaller incisions; better cost-effectiveness. Smaller incisions may also contribute to the reduction of abdominal wall-related complications, such as evisceration and incisional hernias, which ultimately reduces costs due to the elimination of secondary procedures therefore minimizing psychological and surgery-related stress (7). Our study had a favorable complication rate of 16.6% (2 cases), both classified as grade 2 in Clavien-Dindo Classification of Surgery Complications, which is consistent with published literature (Table-3).

**Table 3:** Complications described in literature.

Author	Year	Main Surgery	Open / Lap	N	Clavien I (%)	Clavien II (%)	Clavien IIIa/b	Clavien IV	Total (%)
Inoue (15)	2014	Colorectal	Lap	10	-	2	-	-	2 (20)
Hillings (4)	2008	Colorectal	Open	641	NR	NR	NR	NR	224 (35)
Maurya (9)	2009	Urology	Lap	32	1(3.1)	4(12.5)	-	-	5(15.6)
Reisiger (30)	2005	Urology	Lap	13	1(7.7)	2(15.4)	2(15.4)	-	5(38.4)
Lin (17)	2015	Colorectal	Open	36	-	8 (22)	3 (8.5)	-	11(30.5)
			Lap	36	-	7 (19.4)	2 (5.5)	-	9 (24.9)
Cartapatti (present study)	2015	Urology	Lap	12		2 (16.6)			2 (16.6)

Laparoscopic surgery may also have some disadvantages in comparison to open surgery when evaluating synchronous surgeries. Hemodynamic changes secondary to prolonged pneumoperitoneum and specific patient positioning (for example, Trendelemburg position) could become a limitation to this approach if surgery duration is prolonged. Meininger et al., in a prospective study, evaluated hemodynamic features with patients in the Trendelemburg position with the pneumoperitoneum set at 12mmHg during laparoscopic radical prostatectomy, demonstrating that the head-down position caused only a significant increase in central venous pressure, while the induction of pneumoperitoneum for a period of 4 hours significantly affected the mean arterial pressure. All other hemodynamic parameters remained nearly unaffected (19). Meininger et al. in another study published in 2006 compared overweight and non-obese patients regarding hemodynamics and gas exchange during laparoscopic radical prostatectomy and found severe impairment in oxygen exchange in the overweight group, with no impact in hemodynamics (20). Prolonged pneumoperitoneum can also affect oxidative stress as it raises the intra-abdominal pressure, producing significant organ ischemia followed by reperfusion injury on deflation of the abdomen. This so-called ischemia-reperfusion injury would be present at the end of a prolonged induced pneumoperitoneum and may lead to organ injury and failure (21, 22). Nonetheless, all reported changes were transitory with no permanent impairment in patient's renal or cardio-respiratory functions. Based on these considerations, laparoscopy can negatively impact on patients' health and therefore prolonged surgeries should be avoided, especially in patients with cardio-pulmonary morbidities or obesity. Nevertheless, experienced surgeons can reach acceptable operative time even in combined procedures (9).

Tsivian et al. (18) described 19 patients who underwent concomitant laparoscopic kidney surgery and cholecystectomy and reported an acceptable duration of surgery and hospitalization, in addition to the efficacy and safety of the procedure. Papalia et al. (5) described a series of 32 cases of patients who underwent combined laparoscopic surgery for the treatment of synchronous urological tumors and also found that the technique was feasible and safe, with an acceptable intraoperative duration and level of bleeding and no conversion to open surgery. Gill et al. (23) also described the safety, efficacy and viability of combined surgery in a group of patients with autosomal dominant polycystic kidney disease subjected to bilateral synchronous nephrectomy. When combined laparoscopic surgery involves non-urological diseases, it is important to include other specialists on the surgical team to reduce the duration of the procedure. Moreover, another factor that contributes to achieve this objective is the presence of experienced surgeons (16, 18).

Some authors have also reported cases of robotic-assisted combined laparoscopic surgery, with the same benefits as the combined laparoscopic procedures previously described (24–29). Another important aspect of combined laparoscopic surgery is that it is possible to use the same access sites for both procedures, adding at maximum another one or two entry sites (5, 30).

In tertiary services in countries such as Brazil, where the institution's financial problems sometimes may interfere in treatment election, a combined approach for synchronous pathologies might be a good solution. It seems only logical that one single surgery, minimizing disposable waste, minimizing the total anesthetic and pain medications used, while achieving similar hospitalization periods would be less expensive than the sequential approach. This hypothesis is not confirmed by this study, as analysis of cost-effectiveness was not part of our objectives. Further studies into the cost effectiveness and safety of simultaneous laparoscopic surgeries should be pursued.

## CONCLUSIONS

Combined laparoscopic surgery for the treatment of at least one urological condition is a feasible, viable and safe choice. This approach provides acceptable intra- and postoperative morbidity, has the potential for shorter hospitalization, less anesthetic and pain medication usage, and may decrease recovery time. Because of these potential benefits, this approach can reduce costs and burden for patients. Due to the potential for prolonged operative time, we recommend that this approach should only be done in larger oncologic centers and by experienced surgeons to minimize the risks and optimize its benefits.
